# Tumor‐derived extracellular vesicles activate primary monocytes

**DOI:** 10.1002/cam4.1465

**Published:** 2018-03-30

**Authors:** Kathrin Gärtner, Christina Battke, Judith Dünzkofer, Corinna Hüls, Bettina von Neubeck, Mar‐ kus Kellner, Elena Fiestas, Susanne Fackler, Stephan Lang, Reinhard Zeidler

**Affiliations:** ^1^ Research Unit Gene Vectors Helmholtz Center Munich German Research Center for Environmental Health Munich Germany; ^2^ Department of Otorhinolaryngology Klinikum der Universität (KUM) Munich Germany; ^3^ Department of Otorhinolaryngology University of Duisburg‐Essen Essen Germany

**Keywords:** Extracellular vesicles, immune escape, tumor microenvironment

## Abstract

Tumor cells educate immune effector cells in their vicinity by releasing factors that manipulate their phenotype and function. In fact, the thus generated immunosuppressive tumor microenvironment constitutes an integral part and a hallmark of solid tumors and contributes significantly to tumor development and immune escape. It has long been thought that soluble factors like prostaglandin E2 and TGF‐*β* are the main mediators of these effects. But tumor cells also constantly release large number of extracellular vesicles (EVs), which are important conveyors of immune responses. We show here that tumor‐derived EVs interact with primary monocytes and induce an activated phenotype, which is also observed in tumor‐associated macrophages. Thus, both tumor‐derived EVs and soluble factors together collaborate to form the immunosuppressive milieu of the tumor environment.

## Introduction

Tumors shape their microenvironment to both foster their own survival and growth and to blunt antitumor immune responses. To do so, tumors “educate” associated surrounding cells and co‐opt them to their own benefit. Consequently, the immunosuppressive milieu of the tumor environment is largely attributable to factors, which are released by tumor cells and which directly or indirectly bias the function and phenotype of cancer‐associated fibroblasts, mesenchymal stroma cells, and immune effector cells.

The tumor's immunosuppressive activities of are generally thought to be conveyed by soluble prostaglandin E2 and cytokines like IL‐10 and TGF‐*β* 1 that bind to receptors on target immune cells. However, it is becoming increasingly clear that soluble factors are not the only way how tumors communicate with normal host cells and manipulate their immune functions. Instead, it is firmly established today that tumor cells also constantly secrete large numbers of vesicles of different intracellular origin and composition like exosomes and microvesicles, collectively referred to as extracellular vesicles (EVs) 2. Tumor‐derived EVs (TEVs) became a focus of particular interest to tumor immunologists when it has been shown that they skew the function of cells of the immune system and even promote tumor cell growth and migration 3, 4. For example, TEVs impair lymphocyte responses to IL‐2 5, induce apoptosis in activated T cells 6, down‐modulate the cytolytic activity of NK cells 7, 8, suppress the function of dendritic cells 9, and inhibit differentiation of myeloid cells 10. In essence, TEVs constitute a relevant part of the bidirectional communication between tumor cells and cells in the tumor microenvironment [11, 12 for review].

Thanks to their plasticity, monocytes/macrophages can exert both pro‐ and antitumor activities depending on the environment and the functional program they display. Tumor‐associated macrophages (TAMs), which are suggested to originate from monocytes 13, constitute a pivotal, and usually the most abundant, class of immune effectors of the tumor stroma. TAMs are usually polarized toward the tumor growth and progression promoting M2 phenotype characterized by a constant low‐level release of pro‐inflammatory cytokines on the one hand, and a reduced or defective TNF‐*α* and IL‐12 production and high IL‐10 secretion upon activation on the other side 14.

Cultivation of target cells in conditioned supernatants of permanent cancer cell lines (TuSN) in vitro is a versatile surrogate experimental setting to mimic interactions of secreted tumor‐derived factors with cells of the immune system in vivo 1. Upon cultivation in TuSN, immune cells display an activated phenotype as exemplified by the induction of immune accessory surface markers 15, the release of pro‐ and anti‐inflammatory cytokines 16, and interference with macrophage function 17. It has long been overseen that bioactive TuSN not only contain soluble factors but also large quantities of extracellular vesicles (TEVs) that may contribute to the well‐described immunomodulating activities of TuSN. To this end, we addressed this shortcoming and investigated the interaction of TuSN‐derived TEVs with primary monocytes. Our results show that also TEVs have an important role in that they interact with, and activate, primary monocytes and impinge on their immune function.

## Material and Methods

### Cell culture

FaDu (ATCC‐No. HTB‐43), PCI‐1 (a gift from T. Whiteside, Pittsburgh, PA), and GHD‐1 (established in our laboratory) are human squamous head and neck cancer cell lines, and A549 (ATCC‐No. CCL‐185) is derived from a human adenocarcinoma of the lung. All primary cells and cell lines were maintained in standard cell culture medium (DMEM [Thermo Fisher, Dreieich, Germany] supplemented with 10% heat‐inactivated fetal calf serum [FCS; PAA‐Laboratories, Cölbe, Germany]) at 37°C in a humidified CO_2_ atmosphere. Tumor lines were routinely tested for mycoplasma (Venor^®^ GeM Mycoplasma Detection Kit; Minerva Biolabs, Berlin, Germany). Authentication of the FaDu and A549 cell lines by STR‐PCR has been performed at Eurofins (Ebersberg, Germany). Primary monocytes were isolated from PBMCs from healthy volunteers with CD14‐specific magnetic beads (Miltenyi, Bergisch‐Gladbach, Germany), tested by flow cytometry for the expression of CD14 and CD16 and used directly for further experiments. To study the interaction of monocytes with TEV, 1 × 10e6 monocytes were cultivated in a final volume of 2.5 mL with conditioned supernatants from different tumor cells, with TEV‐depleted supernatants (i.e., supernatants after ultracentrifugation) or isolated TEV (dilution 1:20 in DMEM with 10% FCS).

### ELISA and NF*κ*B‐binding assays

ELISA assays (Mabtech, Nacka Strand, Sweden) and the NF*κ*B p50 transcription factor kit (ThermoFisher) were performed according to the manufacturer's instructions. Briefly, a DNA double‐strand oligonucleotide containing the NF*κ*B consensus sequence was coupled to a ELISA plate, nuclear extracts from primary monocytes were added, and binding of p50 was quantified with a specific primary antibody and a HRP‐coupled secondary antibody, followed by addition of ECL (GE Healthcare, Munich, Germany). Chemoluminescence was measured with a luminometer (PerkinElmer Wallac 1420 Victor2).

### Flow cytometry

The following antibodies were used for flow cytometry: anti‐CD14‐PE (Biolegend, Koblenz, Germany, #367103), anti‐CD16‐FITC (Biolegend, #302005), anti‐EpCAM (clone Ho3; a kind gift of Dr. H. Lindhofer, Munich, Germany). Cells were stained with antibodies diluted in PBS/2% FCS for 15 min and, where applicable, with an appropriate fluorochrome‐labeled secondary antibody for another 15 min. Flow cytometry was performed with a FACSCalibur flow cytometer (Becton Dickinson) and analyzed with FlowJo (Tree Star Inc., Ashland, US).

### Isolation of tumor‐derived extracellular vesicles

Tumor‐derived EVs were isolated from 20 mL conditioned cell culture supernatants as described previously 18. Briefly, cells were grown to approximately 60% confluency, washed twice with PBS, and incubated in DMEM without FCS for 48 h. Then, supernatants were collected and depleted of cells and cellular debris by centrifugation at 450 x g for 10 min and subsequently passed through a 0.45‐μm PES filter. The filtrate was concentrated by ultracentrifugation at 100,000 *g* (4°C) in a Beckman LE‐80K ultracentrifuge in a SW28 or SW32 swing‐out rotor for 2 h. The supernatant was collected and passed through a 0.2‐μm PES filter. This supernatant is here referred to as “TEV‐depleted supernatant.” The pellet obtained after ultracentrifugation was resuspended in 500 μL PBS, and fractions thereof were analyzed for the presence of the EV markers with antibodies against CD63 (antibody generated in our own lab), Alix (Biolegend, #634501), and TSG101 (GeneTex, Irvine, US, #GTX70255) by Western blots and enumerated by NTA as described below. This pellet is here referred to as “tumor‐derived extracellular vesicles” (TEVs). A 1:20 dilution of the isolated TEVs in DMEM with 10% FCS was used for studying the interaction with monocytes. The tumor cell supernatants used throughout this study contained approx. 1–3 × 10e9 EVs per mL as enumerated per NTA, corresponding to a TEV to monocytes ratio of 1000:1.

### Electron microscopy

Droplets of TEVs were placed on formvar‐coated grids, left to adsorb for 1 h and fixed with 2.2% formaldehyde and 0.1% glutaraldehyde for 1 h. After washing with PBS/1% BSA‐C (BIOTREND, Koeln, Germany), grids were blocked with Aurion Block for 30 min. After an additional fixation with 2% glutaraldehyde for 10 min and further washing, grids were stained with 2% phosphotungstic acid for 2 min.

### Nanoparticle tracking analysis (NTA)

Extracellular vesicles were measured by NTA using the ZetaView PMX110 instrument (Particle Metrix, Inning, Germany) calibrated with polystyrene beads of known size and concentration (100 nm NanoStandards; Applied Microspheres, Leusden, The Netherlands). Isolated EVs were diluted in PBS to a concentration of 100–200 particles per video frame. Each sample was measured at eleven positions with three reading cycles at each position. The preacquisition parameters were set to a sensitivity of 70, a shutter of 50, and a frame rate of 30 frames per second. The postacquisition parameters were set to a minimum brightness of 20, a minimum size of 5 pixels and maximum size of 1000 pixels.

### Western blot

Cell lysates were prepared in ice‐cold RIPA buffer with protease inhibitors. Protein concentration was measured by Bradford protein assay (Bio‐Rad), and 10 μg of cell lysate was used for SDS‐PAGE. Gels were electroblotted on a nitrocellulose membrane, followed by blocking for 1 h and an incubation with primary antibodies at 4°C overnight. The membrane was washed and incubated with HRP‐coupled secondary antibodies at room temperature for 1 h and developed with the ECL system (GE Healthcare). The following antibodies were used for immunostaining: mouse anti‐EpCAM (Ho3), mouse anti‐NF*κ*B p65 (MAB5078; R&D Systems, Wiesbaden, Germany), mouse anti‐*α*‐tubulin (Biolegend, #627901), rabbit anti‐p38 (Biolegend, #622401), rabbit anti‐phospho‐p38 (Biolegend, #903501), rabbit anti‐I*κ*B*α* (Cell Signaling, #9242S), and rabbit anti‐phospho‐I*κ*B*α* (Cell Signaling, #2859S). For the preparation of nuclear extracts, cells were isolated by scraping and subjected to the nuclear‐extracts protocol of the NF*κ*B binding assay (Pierce).

### Statistics

Gaussian distribution of values was tested using D'Agostino and Pearson's omnibus normality test. Statistics evaluation was performed using the paired *t*‐test.

## Results

### TEVs interact with primary monocytes

**Figure 1:**
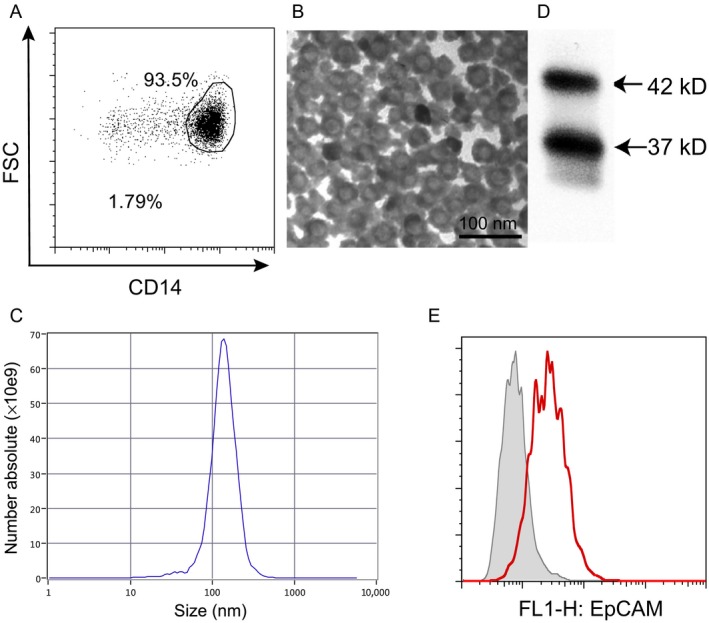
Extracellular vesicles from tumor cells interact with primary monocytes. (A) CD14+ monocytes were enriched from PBMCs by magnetic separation. (B) Electron microscopy of EVs from PCI‐1 cells revealed their typical size and phenotype. (C) NTA analysis of EVs isolated from conditioned TuSN. (D) Immunoblot for EpCAM using lysates from PCI‐1 EVs. Arrows indicate the glycosylated and unglycosylated forms of the proteins. (E) PBMCs were incubated with PCI‐1 EVs for 24 h (red histogram) or in standard DMEM (grey histogram). Binding of TEVs to monocytes was measured by flow cytometry using an EpCAM‐specific antibody.

We first wished to assess whether TEVs interact with primary monocytes. For this, we enriched CD14+ monocytes from PBMCs of healthy donors with microbeads (Fig. 1A). In parallel, we isolated TEVs from the human head and neck cancer cell line PCI‐1 by differential ultracentrifugation. Electron microscopy (Fig. 1B) and nanoparticle tracking analysis (Fig. 1C) revealed that we obtained a pure population of particles of the size and shape typical for extracellular vesicles that also carried the exosomal markers CD63 and TSG101 (data not shown). Because PCI‐1 cells express high amount of the epithelial adhesion molecule EpCAM (not shown) and the molecule has been described to be packaged into extracellular vesicles derived from ovarian cancer cells 19, we next investigated whether TEVs from PCI‐1 also contain EpCAM. An immunoblot revealed that this is indeed the case (Fig. 1D). The distinct signals at 37kD and 42kD are probably due to different glycosylation 20 or proteolytic processing of the protein. In parallel, we tested PBMCs for EpCAM expression and found them to be completely negative (data not show). Thus, EpCAM is a reliable marker for studying the interaction of TEVs with immune effector cells. Therefore, we incubated primary CD14+ monocytes with PCI‐TEVs for 24 h and then stained the cells with an antibody specific for EpCAM as a marker of interaction. Flow cytometry revealed that monocytes clearly became positive for EpCAM after incubation, indicating that they were target cells for PCI‐1 TEVs (Fig. 1E). Similar results were obtained with TEVs isolated from the other tumor cell lines used in this manuscript (not shown).

### TEVs activate the immediate‐early response in monocytes

**Figure 2:**
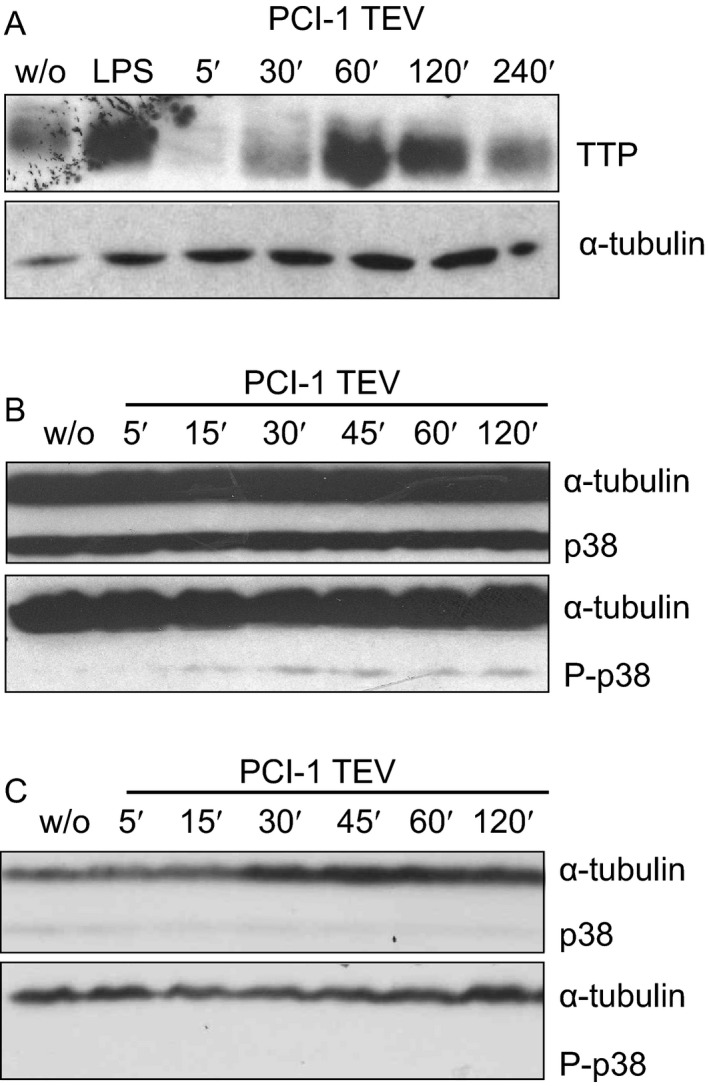
Tumor‐derived EVs induce the phosphorylation of the immediate‐early proteins in primary monocytes. Primary monocytes were incubated with TEVs from PCI‐1 cells and lysates were generated at different time points of incubation. (A) Immunoblots for tristetraprolin (TTP). (B) and (C) Long and short exposure, respectively, of an immunoblot for p38 and phosphorylated P‐p38. A lysate from monocytes stimulated with LPS (1 μg/mL final concentration) was used as a positive control, and tubulin was used as a loading control. w/o = incubation in standard DMEM.

Activation of monocytes is characterized by the rapid increase in biosynthesis and release of pro‐inflammatory TNF‐*α*, which itself is regulated at different levels by immediate‐early genes like tristetraprolin (TTP) and p38 MAPK 21, 22. To investigate whether TEV trigger the immediate‐early response, we incubated primary monocytes with TEVs as described above, generated lysates at different periods of time of incubation and analyzed them by immunoblotting. First, we assessed the expression of TTP, which is a known immediate‐early response gene induced by, for example, toll‐like receptor activation 23 and which has been identified as a major regulator of TNF‐*α* biosynthesis 24. TTP is a zinc finger protein that binds to AU‐rich sequences in the 3′‐untranslated region of various mRNAs including messages for pro‐inflammatory cytokines thereby regulating their stability 24. We observed a transient TTP induction in TEV‐treated monocytes starting at 30 min of incubation and a peak at 1 h of incubation (Fig. 2A). Because TTP function is controlled by p38 MAPK, we next investigated whether TEVs activate the p38 MAPK pathway in primary monocytes. As shown in Figure 2B, phosphorylation of p38 MAPK was evident already after only 15 min of incubation with PCI‐1 TEVs. Together, these data suggest that p38 and TTP are parts of pathways that control TEV‐induced acute activation, as characterized by the release of TNF‐*α*, and function of monocytes.

### TEVs induce the secretion of pro‐inflammatory cytokines by primary monocytes

**Figure 3:**
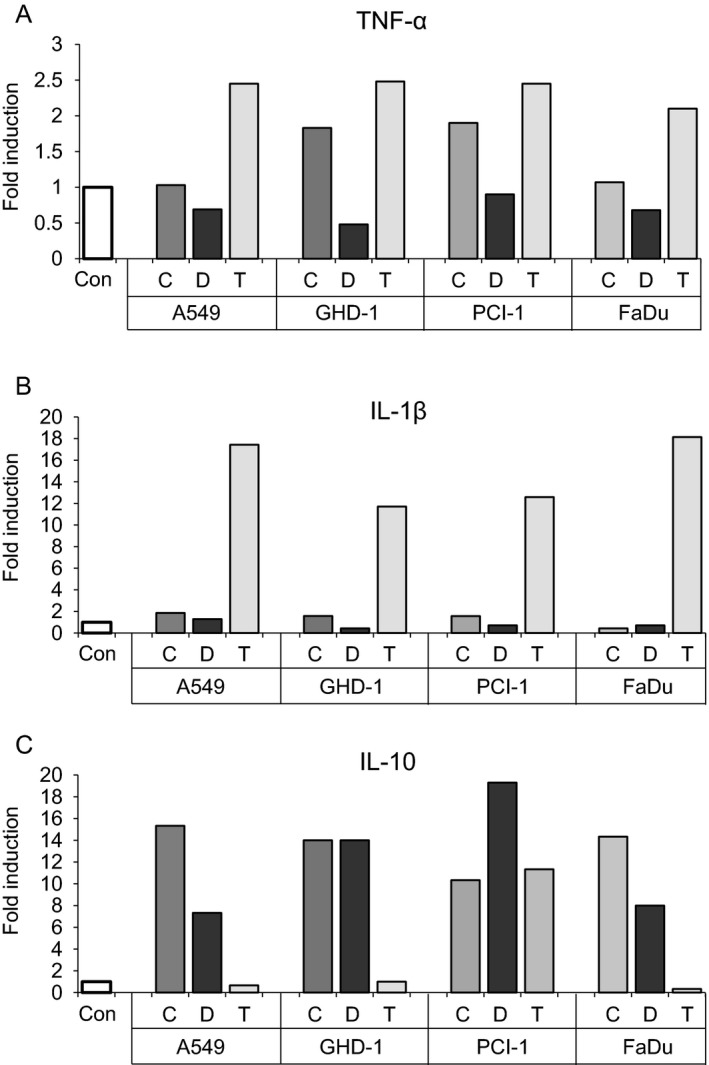
Tumor‐derived EVs induce the secretion of pro‐inflammatory cytokines from primary CD14+ monocytes. Cells were incubated with complete conditioned supernatants (=C) from different cancer cell lines, with TEV‐depleted supernatants (=D) or with isolated TEVs (=T) overnight. As a control, cells were incubated in standard cell culture medium (=Con). The supernatants were tested for cytokines using standard ELISA assays. Values are given as “fold induction” as compared to control cells. Shown is one representative experiment out of three independent experiments.

Constant and low‐level expression of cytokines is typical for tumor‐associated immune cells and characteristic for the smouldering inflammation of the tumor microenvironment 25. In a next series of experiments, we incubated primary CD14+ monocytes either with complete TuSN, EV‐depleted supernatants, or isolated TEVs derived from three head and neck cancer cell lines (PCI‐1, GDH‐1, FaDu) or the lung cancer cell line A549 for 24 h and thereafter measured the concentration of the pro‐inflammatory cytokines TNF‐*α* and IL‐1*β*, and of anti‐inflammatory IL‐10 in the supernatants with ELISA assays. As shown in Figure 3A, both complete TuSN and isolated TEVs from all cell lines tested induced the secretion of pro‐inflammatory TNF‐*α*, while depleted TuSN were much less active. IL‐1*β*, another pro‐inflammatory cytokine, was almost exclusively induced by isolated TEVs. Complete conditioned TuSN revealed only moderate IL‐1*β*‐induction (Fig. 3B). This discrepancy is probably due to the lower number of TEVs in the supernatants as compared to isolated TEVs. Thus, TEVs clearly contribute to the induction of the pro‐inflammatory cytokines TNF‐*α* and IL‐1*β*. In sharp contrast, isolated TEVs did not induce the secretion of detectable amounts of anti‐inflammatory IL‐10, whereas complete and TEVs‐depleted TuSN did (Fig. 3C). These results indicate that CD14+ monocytes are relevant TEVs targets and that IL‐10 is induced by soluble factors present in Tu‐SN in contrast to various pro‐inflammatory cytokines which are more induced by TEVs contained in these conditioned supernatants.

### TEVs activate the NFκB pathway

**Figure 4:**
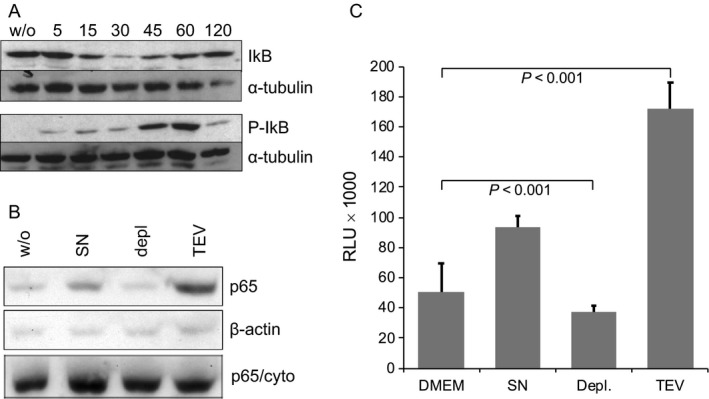
Tumor‐derived EVs activate the NF *κ*B pathway. Primary CD14+ monocytes were incubated with TEVs isolated from conditioned PCI‐1 supernatants. (A) Lysates were generated at different timepoints of incubation and tested for I*κ*B and phosphorylated P‐I*κ*B. Tubulin was used as a loading control. (B) Primary monocytes were incubated with complete PCI‐1 supernatant (SN), in TEV‐depleted SN (depl.) or with isolated TEV for 120 min, nuclei were isolated and the translocation of NF *κ*B‐p65 was tested with an immunoblot. Actin was used as a loading control, and the cytoplasmic fraction (p65/cyto) was used to confirm the specificity of the translocation. (C) Monocytes were incubated as in (B), and the binding capacity of NF *κ*B p50 to the DNA was quantified with a NF *κ*B binding assay kit.

The NF*κ*B signaling pathway is central for monocyte activation but has also been shown to be required for tumor initiation in inflammation‐triggered cancer models 26, 27. Additionally, Hagemann et al. 28 demonstrated that NF*κ*B signaling in TAMs is important for maintaining the M2 phenotype and promoting tumor growth. In a next series of experiments, we therefore investigated whether TEVs also induce the NF*κ*B pathway in monocytes. Activation of NF*κ*B is initiated by the phosphorylation, and subsequent degradation, of I*κ*B 26. We therefore isolated primary CD14+ monocytes as described above, incubated them in the presence or absence of PCI‐1 TEVs for up to 120 min, and then evaluated the phosphorylation status of I*κ*B. As depicted in Figure 4A, phosphorylation was initiated as early as 5 min of incubation and peaked at around 60 min of incubation. Phosphorylation and degradation of I*κ*B liberates NF*κ*B that is subsequently translocated to the nucleus where it binds to NF*κ*B DNA consensus sequences. We therefore incubated primary monocytes as above, isolated the nuclei and performed immunoblots to quantify nuclear NF*κ*B. As shown in Figure 4B, incubation with conditioned PCI‐1 supernatants moderately, while isolated PCI‐1 TEVs strongly induced the translocation of NF*κ*B to the nucleus. In contrast, TEV‐depleted PCI‐1 supernatants did not induce translocation (Fig. 4B). In line with these results, PCI‐1 also strongly induced the binding of NF*κ*B p50 to the DNA (Fig. 4C), indicative for its activity as a transcription factor.

## Discussion

The suppressive tumor environment significantly contributes to tumor initiation and progression. It is known that tumor educates immune effector cells in the tumor parenchyma to contribute to an immunosuppressive milieu and to foster tumor development. It is also known that tumor cells release immunosuppressive soluble factors like prostaglandin E2 and TGF‐*β* to interfere with the phenotype and function of immune effector cells which, in turn, contribute to tumor growth and progression. Because tumor cells also constantly release high numbers of extracellular vesicles (TEVs), we wished to investigate their impact on primary monocytes, as tumor‐associated macrophages (TAMs) constitute a major, monocyte‐derived population of the tumor infiltrate with mainly tumor‐promoting properties 13.

As has been demonstrated by others, conditioned supernatants from tumor cells induce the secretion of various cytokines in primary monocytes. Here, we decipher this interaction in more detail and show that the two major constituents of these supernatants, soluble factors, and TEVs, contribute differentially to these effects: While TEVs interact with primary monocytes and largely induce a pro‐inflammatory phenotype as characterized by the secretion of pro‐inflammatory IL‐1*β* and TNF‐*α*, soluble factors in TEV‐depleted supernatants counteract this activated phenotype by triggering monocytes to secrete immunosuppressive IL‐10. Collectively, soluble factors and TEVs together induce in monocytes a phenotype reminiscent to TAMs. This so‐called M2 phenotype is characterized by a chronic, sub‐optimal activation, a reduced production of pro‐inflammatory cytokines, and enhanced secretion of anti‐inflammatory cytokines like IL‐10.

Despite the fact that throughout the last years numerous studies have characterized the composition, phenotype, and possible function of TEVs in vitro, little is known about their biology in vivo. This discrepancy is largely owed to the fact that it is challenging to detect and follow TEVs in vivo and also that it is demanding recapitulating the chronic interaction between TEVs and nontumor cells in their vicinity in cell culture. This holds also true for some of our experiments. Although we show that TEVs interact with, and activate, primary monocytes, it remains to be clarified how this interaction and activation takes place, that is, whether binding of TEVs is sufficient or whether TEVs have to be engulfed to stimulate dedicated intracellular receptors.

Taken together, we show that tumor cells interact with cells of the tumor microenvironment on various layers and that TEVs significantly contribute to this interaction. Although it is hard to recapitulate and decipher the complex tumor host cell interaction in vitro, we believe that the immunological relevance of TEVs is considerable, given their constitutive secretion and high local concentration. Future research should aim at characterizing the impact of TEVs onto other cellular constituents of the tumor environment more extensively (including a more comprehensive cytokine profile) and at better understanding the molecular basis underlying this interaction.

## Conflict of Interest

The authors declare no conflict of interest.
